# Structural New Data for Mitochondrial Peroxiredoxin From *Trypanosoma cruzi* Show High Similarity With Human Peroxiredoxin 3: Repositioning Thiostrepton as Antichagasic Drug

**DOI:** 10.3389/fcimb.2022.907043

**Published:** 2022-07-06

**Authors:** Lucio Rivera-Santiago, Ignacio Martínez, Ruben Arroyo-Olarte, Paulina Díaz-Garrido, Roberto I. Cuevas-Hernandez, Bertha Espinoza

**Affiliations:** Departamento de Inmunología, Instituto de Investigaciones Biomédicas, Universidad Nacional Autónoma de México, Ciudad de México, México

**Keywords:** *Trypanosoma cruzi*, mitochondrial peroxiredoxin, bioinformatic analysis, repositioning Thiostrepton, trypanocidal activity

## Abstract

*Trypanosoma cruzi*, the causal agent of Chagas disease, has peroxiredoxins (PRXs) expressed in all stages of the parasite and whose function is to detoxify oxidizing agents, such as reactive oxygen species (ROS). These proteins are central for the survival and replication of the parasite and have been proposed as virulence factors. Because of their importance, they have also been considered as possible therapeutic targets, although there is no specific drug against them. One of them, the mitochondrial PRX (TcMPX), is important in the detoxification of ROS in this organelle and has a role in the infectivity of *T. cruzi*. However, their structural characteristics are unknown, and possible inhibitors have not been proposed. The aim was to describe in detail some structural characteristics of TcMPX and compare it with several PRXs to find possible similarities and repositioning the antibiotic Thiostrepton as a potential inhibitor molecule. It was found that, in addition to the characteristic active site of a 2-cys PRX, this protein has a possible transmembrane motif and motifs involved in resistance to hyper oxidation. The homology model suggests a high structural similarity with human PRX3. This similarity was corroborated by cross-recognition using an anti-human PRX antibody. In addition, molecular docking showed that Thiostrepton, a potent inhibitor of human PRX3, could bind to TcMPX and affect its function. Our results show that Thiostrepton reduces the proliferation of *T. cruzi* epimastigotes, cell-derived trypomastigotes, and blood trypomastigotes with low cytotoxicity on Vero cells. We also demonstrated a synergic effect of Thriostepton and Beznidazol. The convenience of seeking treatment alternatives against *T. cruzi* by repositioning compounds as Thiostrepton is discussed.

## Introduction

The protozoan *T. cruzi* is the etiologic agent of the Chagas disease (American trypanosomiasis), one of the major causes of morbidity and mortality in many countries of Latin America. Because of human migrations, Chagas disease is emerging in other regions (Europe and the United States principally), and it is estimated that 6 to 7 million people are currently infected worldwide (WHO, 2021). *T. cruzi *infects two hosts, the insect vector, a member of the Reduviidae family, and mammals such as humans. During its life cycle within these hosts, the parasite faces oxidant stress. When a vector feeds on a mammal infected with blood trypomastigotes, they travel to the posterior intestine. Large amounts of hemoglobin produced by food are degraded to heme (Nogueira et al., 2015), increasing the production of reactive oxygen species (ROS), which can eliminate the parasite. Another critical component in generating ROS within the vector is the prophenoloxidase present in hemolymph and hemocytes. It participates in melanization, phagocytosis, and encapsulation of parasites (Machado-Silva et al., 2016) and the production of intermediates of ROS and reactive nitrogen species. When the vector feeds on its host, it detects near the suction area, and the host can enter the parasite due to micro-abrasions in the skin caused by scratching. Once *T. cruzi* enters to the host, it interacts with several elements of the immune response like macrophages or neutrophils, which can potentially eliminate it through ROS such as superoxide anion (O^2.−^), hydrogen peroxide (H_2_O_2_),_ _and peroxynitrite (ONOO^−^). The host–parasite relationship has resulted in that *T. cruzi* developed effective mechanisms of evasion and resistance to the immune system as well as efficient antioxidant machinery. The peroxiredoxins (PRXs) are proteins responsible for inactivating ROS, organic peroxides, peroxynitrites, and peroxynitrous acid, which generate an oxidizing environment for the parasite (Perkins et al., 2015).

In *T. cruzi*, five PRXs that are found in specific organelles have been reported: cytosolic PRX (TcCPX), mitochondrial PRX (TcMPX), ascorbate-dependent heme peroxidase present in the endoplasmic reticulum (TcAPX), glutathione peroxidase I present in the glycosome (TcGPXI), and glutathione peroxidase II present in the endoplasmic reticulum (TcGPXII) (Wilkinson et al., 2000).

The mitochondrial enzyme TcMPX is one of the most studied, and it belongs to the typical 2-Cys PRXs, with a molecular weight of ~25 kDa. TcMPX concentrates on the kinetoplast, indicating that its main function is to protect the mitochondrial genome from peroxide-mediated damage (Wilkinson et al., 2000). Furthermore, this enzyme interacts with other molecules such as tryparedoxin II during oxidative stress conditions (Dias et al., 2018). It has been demonstrated that the overexpression of TcMPX protects *T. cruzi* against a wide range of peroxynitrites derived from immune cells and increases the resistance to H_2_O_2_, corroborating its participation as an antioxidant defense mechanism (Piacenza et al., 2008). In addition, during *T. cruzi* differentiation, there is an increase of TcMPX expression in trypomastigotes (infective stage) compared with epimastigotes (non-infective stage), analyzed in several *T. cruzi* strains (Piacenza et al., 2009). Furthermore, there is a correlation between virulence and the expression levels of these proteins, which could facilitate the establishment of the parasite in the host and resistance to drugs such as Nifurtimox (NF) (Piñeyro et al., 2008; Specker et al., 2022). Finally, TcMPX has a role as a partner of several proteins important for cellular metabolism (Peloso et al., 2016). Therefore, TcMPX has been proposed as an attractive candidate for the development of anti–*T. cruzi* drugs (Wilkinson et al., 2000). However, to date, no studies have been published addressing this possibility *in vivo*, *in vitro*, or *in silico*. In the present work, TcMPX from two *T. cruzi* strains were sequenced and a phylogenetic study was done. An *in silico* analysis was also carried out, and TcMPX structural properties were deduced from a model generated by homology. Finally, the role of a potential inhibitor of TcMPX on the proliferation of *T. cruzi*, cytotoxicity on Vero cells, and synergic effect with Beznidazol (BZ) were evaluated, and its possible role as a therapeutic drug is discussed.

## Materials and Methods

### Parasites and DNA Extraction

Epimastigotes of Mexican TcI Qro (TBAR/MX/0000/Queretaro) and Ninoa (MHOM/MX/1994/Ninoa) strains were cultured as previously described (López-Olmos et al., 1998). Cell-derived trypomastigotes of Qro strain were obtained from Vero cells previously infected as described (Rodríguez-Hernández et al., 2019). Infected cells were cultured in 75-cm^2^ flasks instead using Dulbecco's Modified Eagle's Medium (DMEM), pH 7.2, plus 10% Fetal Bovine Serum (FBS), glutamine (2 mg/ml), penicillin (100 units/ml), and streptomycin (100 mg/ml), in a humidified 5% CO_2_ atmosphere at 37°C. For maintenance, confluent cultures cells were washed with 5 mM Ethylenediaminetetraacetic acid (EDTA), incubated for 5 min with trypsin (1 mg/ml), diluted, and re-plated. Blood trypomastigotes were obtained from Balb/c mice infected with the Qro strain. Maintenance was performed by bleeding mice every 18–20 days and infecting healthy 8-week-old female mice with 1 × 10^5^ parasites intraperitoneally. Genomic DNA extraction of epimastigotes was performed with the phenol-chloroform technique, using 20 ml of logarithmic phase parasite culture (60 × 10^6^ parasites/ml) (Macedo et al., 1992). Nucleic acid integrity was analyzed by 1% agarose gel electrophoresis, and its concentration and purity were assessed using a NanoDrop 1000 (Thermo Fisher Scientific, Waltham, MA, USA). The DNA was kept at −20°C until used.

### PCR and TcMPX Sequencing

The Polymerase Chain Reaction (PCR) to amplify TcMPX was performed using 200 ng of DNA, and specific primers were designed using the software *Primer1* (http://primer1.soton.ac.uk/primer1.html): TcMPX-Fw (5′-ATGTTTCGTCGTATGGCCG-3′) and TcMPX-Rv (5′-TCATGCGTTTTTCTCAAAATATTCA-3′). The conditions employed were as follows: 5 U Platinum™ Pfx enzyme (Invitrogen, Massachusetts, USA), 10 mM of each primer, and 4 mM MgCl_2_. One initial step of 94°C for 3 min, 35 cycles (30 s at 94°C, 30 s at 60°C, and 45 s at 68°C), and a final elongation step (5 min at 68°C). The amplicons obtained (330-bp fragment) were purified using the innuPREP DOUBLEpure Kit (Analytik Jena, Jena, Germany). They were sequenced in the Laboratorio de Secuenciación Genómica de la Biodiversidad y de la Salud (UNAM) with the Sanger technique, using a 3500xL genetic analyzer (Applied Biosystems, Massachusetts, USA). The obtained sequences for Qro and Ninoa were analyzed using the software Chromas. The consensus sequence was determined using the software Bioedit. Finally, the two sequences were uploaded to the GenBank as QroMPX (accession number QKE53461.1) and NinoaMPX (QKE53460.1).

### Phylogenetic and Comparative Analysis

The molecular weight, isoelectric point, and net charge were determined using the software Pepstats 6.6.0 (http://www.ebi.ac.uk/Tools/services/web). The conserved domain-based prediction was performed with the online software SMART (Simple Modular Architecture Research Tool) (http://smart.embl-heidelberg.de/). For the comparative analysis, several sequences of mitochondrial PRXs from different organisms were obtained from GenBank and Protein Data Bank (PDB) (**Supplementary Table 1**). The alignment of the sequences was performed using the software ClustalW. The phylogeny was established by Neighbor-Joining (NJ) analysis, and the bootstrap statistical method (10,000 replicates) was performed with MEGA 7.0. The calculation of the genetic distances was carried out by p-distance. Only the positions with coverage greater than or equal to 95% were considered. The resulting phylogenetic trees were visualized with FigTree V1.4.3 (http://tree.bio.ed.ac.uk/software/figtree). For the identification of conserved amino acids residues and functional motifs, multiple alignments of PRXs were performed using T-coffee (https://www.ebi.ac.uk/Tools/msa/tcoffee/). The prediction of transmembrane helices in QroMPX proteins was done using TMHMM 2.0 software (https://services.healthtech.dtu.dk/service.php?TMHMM-2.0).

### TcMPX Homology Modeling

The homology modeling of QroMPX was performed by two different platforms: SWISS-MODEL (https://swissmodel.expasy.org/interactive) and Phyre2 (http://www.sbg.bio.ic.ac.uk/phyre2/). The resulting models were visualized and compared with PyMol Molecular Graphics System version 2.4.0 (Janson et al., 2017). In addition, the steric arrangement of the amino acids residues and the reliability of the protein structure was validated by the software Procheck, whereas the general rate of error frequencies was established using the software ERRAT. Both software are included in the package SAVES v6.0 (https://saves.mbi.ucla.edu/).

### Immunodetection by Western Blot Using a Commercial Antibody

As described above, epimastigotes of *T. cruzi* Qro strain were cultured for four days. The parasites were harvested and heat-lysed at 100°C for 5 min in lysis buffer (12% Sodium Dodecyl Sulfate (SDS), 10 mM Hepes, pH 7.0). The protein was quantified with the DC Protein Assay Kit (Bio-Rad, California, USA). Then, 12 µg of the protein were separated by electrophoresis in a 12% SDS-Polyacrylamide Gel Electrophoresis (PAGE) and blotted to nitrocellulose membrane. Western blot (WB) analysis was performed by anti-PRX IgG1 B-11 (1:2,000) (137222, Santa Cruz Biotechnology, Texas, USA) as primary antibody and anti-Mouse IgG-Peroxidase (1:4000) (A-9044, Sigma Aldrich, Missouri, USA) as a secondary antibody. The WB was revealed by the addition of 3,3-diaminobenzidine (D5637, Sigma Aldrich, Missouri, USA).

### Molecular Docking With TS

To explore the possible interaction between QroMPX and Thiostrepton (TS), we performed an *in silico* molecular docking study using AutoDock Vina (ADV) 1.1.2 on Mac Os X System (Trott and Olson, 2010). The receptors used for this analysis were the QroMPX model obtained by homology and human PRX3 (PDB 5JCG). Receptors and TS ligand (PDB ID: 2L2W) were prepared and converted to *.PDBQT files in AutoDockTools 1.5.6 (Sanner, 1999). Protein preparation was performed using a standard protocol consisting of the removal of co-crystallized ligands and water molecules; polar hydrogens were added, and Kollman charges for all receptor atoms were computed to assess hydrogen-bonding interactions. All the other parameters were kept at their default settings. The TS ligand was docked at each receptor using a grid box: 60 Å × 60 Å × 60 Å centered at the midpoint between the Cα of the peroxidatic and resolutive cysteines (C_P_ and C_R_, respectively). That is, grid center for PRX3: X = 5.865 Å, Y = −27.873 Å, and Z = −27.243 Å; and grid center for QroMPX: X = 23.17 Å, Y = 23.233 Å, and Z = −3.844 Å. The docking algorithm of ADV was used to find the best complex between ligand and protein. A maximum of 20 conformers were considered for each ligand, and the complex with the most favorable free binding energy was selected for analyzing the interactions and the binding mode between the PRXs and TS with MGLTools 1.5.6 and Discovery Studio Visualizer v21 software.

### TS Activity on *T. cruzi*

TS (Sigma, T8902), a human PRX3 inhibitor used in anti-cancer therapies, was tested for its ability to *T. cruzi* damage. Epimastigotes (2 × 10^6^) were seeded in 96-well plates (200 µl per well) and incubated at 28°C in the presence or absence of several concentrations of TS (1–40 µM) for 24 and 48 h. Benznidazole (BZ, 25 µM) or vehicle (Dymethyl sulfoxide (DMSO) 0.5%) were included as positive and negative controls, respectively. Then, the parasite number in the culture was counted in a Neubauer hemocytometer and reported as a growth percentage. Each condition was evaluated in duplicate in three independent assays. Analysis by one-way ANOVA with Tukey’s multiple comparison was done using GraphPad Prism version 5 for Windows. The mean lethal concentration (IC_50_) of TS to eliminate 50% of the parasites present in the culture was calculated as previously described (Martínez et al., 2013).

Supernatants containing cell-derived trypomastigotes were collected from Vero cells infected with Qro strain by centrifugation at 3,000×g for 20 min and resuspended in complete DMEM medium (Invitrogen, USA). Parasites were counted by microscopic observation using a Neubauer hemocytometer and adjusted to 2 × 10^6^/ml. Parasites were seeded (100 μl per well) in duplicates in a 96-well microplate, in presence or absence of several concentrations of TS and BZ as above, and incubated at 37°C, with 5% of CO_2_ for 24 h as reported previously for other compounds (Adessi et al., 2022). Parasite number was counted as described above. Each condition was evaluated in duplicate in two independent assays. Blood trypomastigotes were obtained as described in Section 2.1. Parasites were counted and blood was diluted with DMEM medium to reach 2x10^6^ trypomastigotes/ml as described previously (Sánchez Alberti et al., 2022). These parasites were incubated with TS, like cell-derived trypomastigotes. Parasite number was counted as described above and reported as the percent of parasites. Each condition was evaluated in duplicate in three independent experiments. Statistical analysis was done as reported for epimastigotes.

### Cell Cytotoxicity Assay on Vero Cells

Vero cells were maintained in DMEM medium supplemented with 10% FBS and reseeding 1/6 of the total culture content every third day. Cytotoxicity produced by TS was evaluated using 3-[4,5-dimethylthiazol-2-yl]-2,5 diphenyl tetrazolium bromide (MTT) assay. Briefly, Vero cells were seeded in a 96-well plate (5,000 per well) and allowed to adhere for 12 h. Then, the medium was removed, and 200 µl of DMEM was added in the absence or presence of TS at concentrations of 2.5 to 40 µM for further 24 h. Next, the medium was removed, and three washes with PBS were performed. Then, 200 µl of DMEM was added in the presence of MTT at 0.8 mg/ml (Sigma, USA) and incubated for 4 h at 37°C. The medium was removed, and the formazan salt was solubilized using 120 µl of DMSO. Absorbance was recorded in a microplate reader with a wavelength of 595 nm and a filter of 655 nm as reference. Three independent assays with duplicates of each condition were performed. Data were plotted and analyzed by one-way ANOVA using GraphPad Prism software.

### Synergy Effect Between TS and BZ

To evaluate the synergistic effect of TS and BZ, first, we calculated the BZ IC_50_ on cell-derived trypomastigotes; we incubated the parasites with several concentrations of BZ (0.39–50 μM) for 24 h. The IC_50_ was calculated as 0.96 ± 0.04 (**Supplementary Figure 3**). Then, we used the BZ IC_50_ in synergy assays with TS. Cell-derived trypomastigotes (2 × 10^5^) were seeded in 96-well plates (100 µl per well) and incubated at 37°C with 5% of CO_2_ in the presence or absence of several concentrations of TS (1.25–10 µM) and BZ at 1.0 µM for 24 h. Parasites in the culture were counted in a Neubauer hemocytometer and reported as parasite percent. Three independent assays with duplicates of each condition were performed. Using GraphPad Prism software, data were plotted and analyzed by non-linear regression with Kolmogorov–Smirnov posttest.

## Results

### QroMPX and NinoaMPX Sequences Analysis

**Figure 1:**
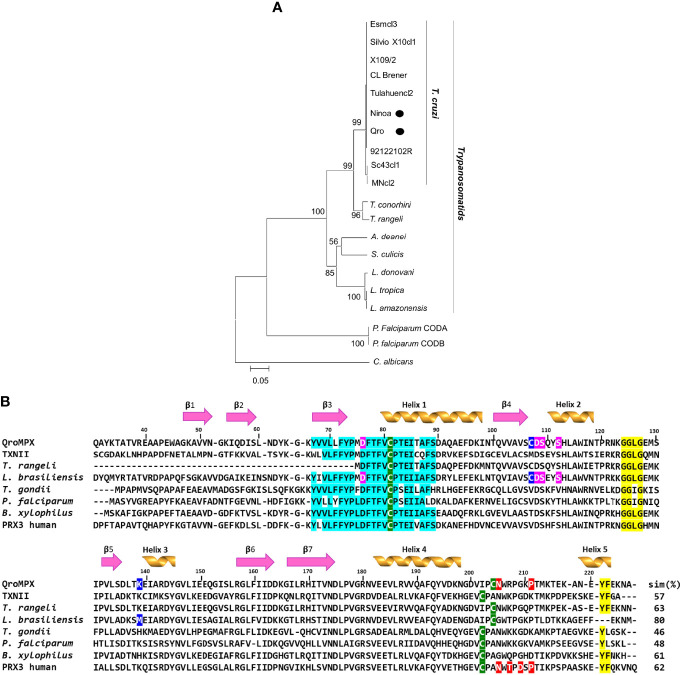
Phylogeny of QroMPX and NinoaMPX and alignment with other mitochondrial PRXs. **(A)** The phylogeny was inferred using the Neighbor-Joining method. The percentage of replicate trees in which the associated taxa clustered together in the bootstrap test (10,000 replicates) are shown next to the nodes. The evolutionary distances were computed using the p-distance method and are in the units of the number of amino acid differences per site (scale bar). The analysis involved 20–amino acid sequences. All positions with less than 95% coverage were eliminated. The sequences of TcMPX from Mexican strains Qro and Ninoa (●) were grouped in the clade of trypanosomatids, close to other T. cruzi strains. **(B)** The sequences were aligned as indicated in materials and methods. Beta sheets (1–7) and a-helix are indicated on the corresponding sequence. The numbering corresponds to the position of the amino acids in the sequence of QroMPX. The cysteines of the catalytic site are indicated in green background. The motifs involved in susceptibility (yellow background) and resistance (red background) to hyperoxidation are also indicated. The transmembrane sequence identified in *B. xylophilus* (ABW81468.1) and the identical amino acids in the other sequences are indicated in cyan. The amino acids that interact with divalent cations (pink background) and those that stabilize the binding (blue background) identified in *L. brasiliensis* (XP_001562236.1) are also indicated in QroMPX. The first 30 amino acids of the QroMPX and *L. brasiliensis* sequences were cut to facilitate alignment. The other sequences used were from *T. cruzi* (TXNII, PDB 4LLR), *T. rangeli* (ESL05855.1), *T. gondii* (AAG25678.2), *P. falciparum* (BAA97121.1), and human PRX3 (PDB 5JCG). At the end of each sequence, the percentage of similarity (sim%) with QroMPX is indicated.

The QroMPX and NinoaMPX have the same nucleotide sequences and do not show differences in their protein sequences (data not shown). Both TcMPX have a predicted size of 226 aa, a molecular weight of 25.5 kDa, an isoelectric point of 7.65, and a net electric charge of 2. A NJ analysis was performed to establish the phylogeny of both proteins. The topology of the obtained tree shows that both sequenced proteins belong to the trypanosomatids clade. Both protein sequences are very similar to the other from *T. cruzi* because tree topology shows a comb structure, characteristic of sequences that are highly similar to each other (**Figure 1A**).

### Multiple Alignments and Functional Motifs Identification

Because of the high identity between QroMPX and NinoaMPX, we decided to work only with the QroMPX as a representative molecule. Multiple alignments were performed with other MPX (including the best-known human PRX3) to establish the presence of functional motifs in the sequence. This analysis showed a percentage of 48%–63% of similarity between QroMPX and the other proteins. In addition, the peroxidatic cysteine (Cp81) and resolving cysteine (C_R_204) from the catalytic site are almost the same in all PRX. However, the second catalytic site in QroMPX has two amino acid changes (IPC instead of VCP). This is one of the notable differences between QroMPX and the myochondrial tryparedoxin TXNII from *T. cruzi* that does preserve the VCP sequence at the C-terminus. Adjacent to the C_R_ (C204) are present two amino acids (N205 y P211) and GGLG and YF motifs, involved in events of hyper oxidation on several PRXs (**Figure 1B**).

### QroMPX Homology Modeling

**Figure 2:**
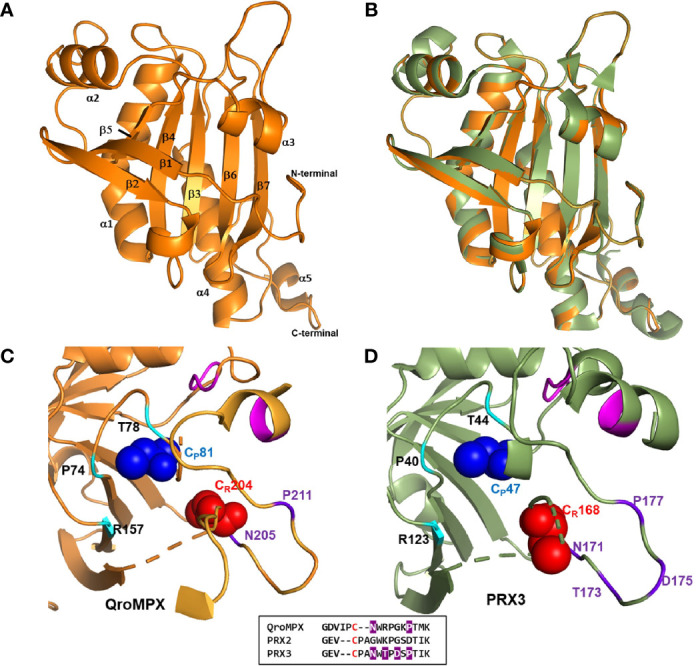
QroMPX tertiary structure model. Modeling was performed as indicated in Materials and Methods. **A)** Monomer of the protein. Alpha-helices and beta sheets are indicated with the corresponding numbering. **B)** Overlap of QroMPX (orange) and human PRX3 (PDB 5JCG, green). **C)** The amino acids surrounding the peroxidatic (CP) and resolutive (CR) cysteine are shown for QroMPX. The GGLG and YF motifs are indicated in pink. The catalytic triad (PTR) is indicated in cyan. The amino acids that could participate in the resistance to hyper oxidation are shown in purple. The comparison of these amino acids in the sequences of QroMPX (QKE461.1), human PRX2 (ABB02182.1), and human PRX3 (PDB 5JCG) are shown in the lower box (in red resolutive cysteines, in purple amino acids involved in resistance to hiperoxidation). **D)** The position of the amino acids around the catalytic site in human PRX3 are shown with the same colors as in C.

The models generated by homology in the two platforms used were very similar (**Supplementary Figure 1A**). Furthermore, a similarity was observed between the amino acid sequence Y67 to S89 of QroMPX with a transmembrane motif described in *B. xylophilus*. Because of this, an analysis was carried out to establish the probability that QroMPX is a transmembrane protein, and the probability is 0.014. Therefore, there is no enough evidence to say that QroMPX can be a transmembranal protein under this analysis. The validation of the model using the Ramachandran plot showed that 90.2% of the amino acids are in the favorable region, indicating good reliability of the structure. The ERRAT plot showed a quality factor of 95.7%, indicating a good model quality. Thus, the model was representative with seven parallel and antiparallel β-sheets, and five α-helices of different lengths (**Figure 2A**). The resulting modeling in all the servers suggests that the template for our target protein (QroMPX) was the human PRX3. The structural similarity between both proteins is highlighted when they are overlapped (**Figure 2B**). The molecular modeling showed that QroMPX monomers could interact forming an interface type B to generate homodimers (**Supplementary Figure 2A**). They could be formed by the interaction of the β7 sheets from each subunit (**Supplementary Figure 2B**). By assembling five dimers, the formation of oligomers (decamers) was also modeled (**Supplementary Figure 2C**), and the electric charges in the oligomer show that the negative charges are preferably distributed inside the structure (**Supplementary Figure 2D**) as reported in other PRXs.

The C_P_ (C81) from a QroMPX monomer could be associated with the C_R_ (C204) from the adjacent monomer. In this conformational structure, C_P_ is surrounded by the catalytic triad formed by the amino acid residues P74, T78, and R157, close to the motifs GGLG and YF, whereas C_R_ is close to the amino acid residues N205 and P211 (**Figure 2C**). The location of the amino acid around the catalytic site is very similar to the human PRX3 (**Figure 2D**).

### Similarity Between QroMPX and PRX3

**Figure 3:**
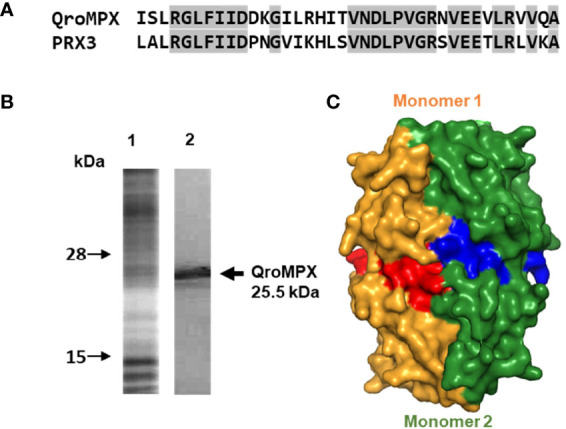
Recognition of QroMPX by anti-human PRX3 antibody. **(A)** Shared sequence between QroMPX and PRX3 recognized by a commercial antibody. Identical amino acids are shaded. **(B)** Western blot was performed as indicated in materials and methods. The total parasite protein transferred (lane 1, Coomassie Blue stain) and the recognition of QroMPX by the commercial anti-PRX antibody (lane 2). **(C)** Model showing the regions on the surface of each QroMPX monomer (red and blue) that can be recognized by the antibody.

Because of the observed similarity between human PRX3 and QroMPX, it was decided to test whether a commercial anti-PRX antibody that recognizes the former could also recognize the *T. cruzi* protein because the amino acid sequence recognized by the commercial antibody is very similar in both proteins (**Figure 3A**). The commercial antibody recognized in a *T. cruzi* total protein extract a protein with molecular weight close to that predicted from TcMPX (**Figure 3B**). Interestingly, when modeling the recognition site in the QroMPX dimer, it was observed that a region of the sequence is exposed on the surface of the molecules, which could facilitate the recognition of these structures by commercial antibodies (**Figure 3C**).

### TS-QroMPX Docking

**Figure 4:**
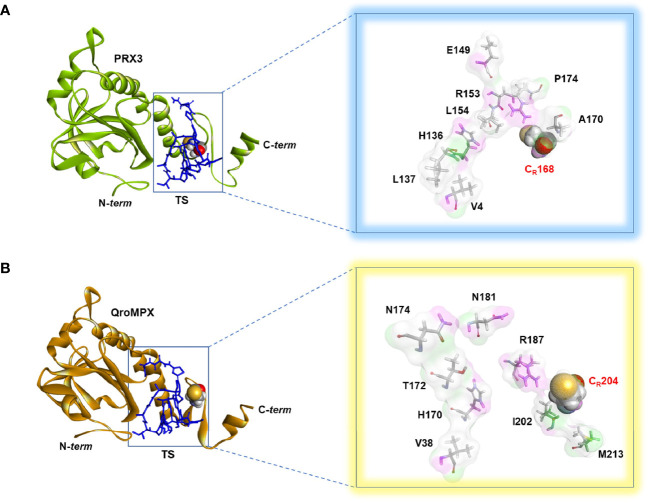
TS-PRX3 and TS-QroMPX Docking. **(A)** Binding mode between TS (blue sticks) and human PRX3 (green). The amino acids (sticks format) and cysteine resolutive (ball format) involved in the interaction are shown in the box. **(B)** Binding mode between TS (blue sticks) and QroMPX (orange). The amino acids (sticks format) and cysteine resolutive (ball format) involved in the interaction are shown in the box.

Because QroMPX and human PRX3 are structurally similar, there is a possibility that QroMPX was susceptible to PRX3 inhibitors such as the antibiotic TS. To explore this hypothesis, we performed an in silico molecular docking study. Our computational model considered a stable conformation of minimum energy for the protein QroMPX obtained by homology and for PRX3 obtained from the PDB (5JCG). To eliminate bias from the rigid nature of the receptor in our model, we used one monomer of each protein for ligand-receptor interaction analysis, because the ligand (TS) would be physically hindered by its bulk. Therefore, our results showed that TS has an affinity for the catalytic site, particularly, the resolutive-cysteine ​​(C_R_) in PRX3 (**Figure 4A**). Something similar was observed in the TS-QroMPX interaction model in which, in addition to the resolutive cysteine, the amino acids V38, H170, T172, N174, N181, R187, I202, CR204, and M213 are involved, with a ΔG = 7.0 kCal/mol (**Figure 4B**). These results suggest that TS has a high probability of binding to cysteines in the catalytic domain of QroMPX and interfering with their biological activity.

### TS Activity on *T. cruzi*

**Figure 5:**
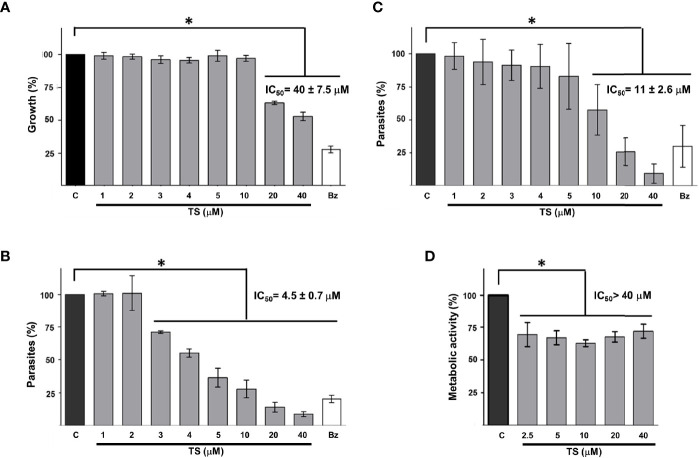
Tripanocidal effect of TS. Epimastigotes **(A)**, cell-derived trypomastigotes **(B)**, blood trypomastigotes **(C)**, and Vero cells cytotoxicity **(D)** were incubated in the presence or absence of TS at the indicated micromolar concentrations. Parasite number was established by counting at 48 h **(A)** or 24 h **(B, C)** of incubation. Metabolic activity was measured using MTT assay at 24 h **(D)**. The results are presented as media ± standard deviation. Bz (25 μM) was used as a positive control. Media without TS **(C)** was used as control. Statistically significant differences are indicated with an asterisk (p < 0.05).

To establish whether TS could reduce the growth of *T. cruzi* as it does with some cancer cells, epimastigotes were incubated in the presence of this compound for 24 and 48 h. It was observed that TS reduced the number of parasites only at 48 h of incubation at concentrations of 20 and 40 µM (**Figure 5A**). The IC_50_ value was established at 40 ± 7.5 μM.

It was observed that TS has a high trypanocidal effect on the infective state of *T. cruzi* because, at 24 h, more than 50% of the cell-derived trypomastigotes had been eliminated with less than 5 μM of the compound (**Figure 5B**). The IC_50_ value was calculated at 4.5 ± 0.7 μM. On the other hand, blood trypomastigotes were also sensitive to the effect of TS because, with 20 μM of TS, more than 65% of the parasites were eliminated from the culture (**Figure 5C**). The IC_50_ value was calculated at 11 ± 2.6 μM. No morphological damage was observed at optical microscopy. Preliminary experiments show no effect on intracellular amastigotes at 24 h of incubation with several TS concentrations.

### Cytotoxicity Effect of Ts on Vero Cells

A significant decrease in the metabolic activity of Vero cells was observed in the presence of TS, but this was not greater than 25%, even at the highest TS concentration used (40 µM). The IC_50_ for Vero cells is greater than 40 µM, but it could not be established, so the selectivity index could not be calculated. The cytotoxicity of TS on Vero cells was moderate since between 62% and 72% of the cells maintained their metabolic activity (**Figure 5D**).

### Synergic Effect Between TS and BZ

**Figure 6:**
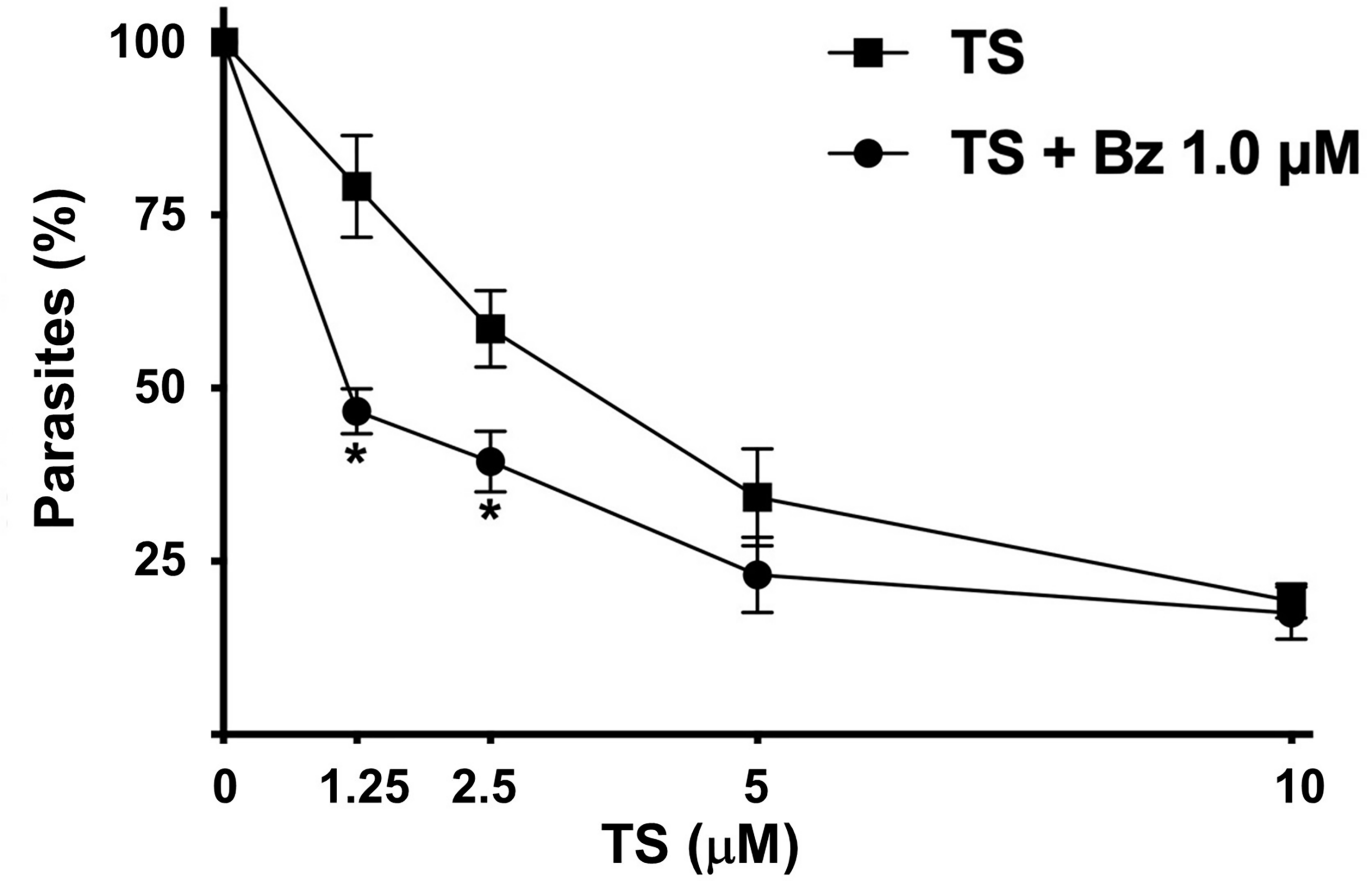
Synergic effect of TS and Bz. Cell-derived trypomastigotes were incubated in the presence of different concentrations of TS alone or in combination with 1.0 μM Bz. Parasite number was established by counting in a Neubauer chamber after 24 h of drugs incubation. The results are presented as media ± standard deviation. Statistically significant differences are indicated with an asterisk (p < 0.001).

Because of the trypanocidal effect of TS on *T. cruzi* trypomastigotes, we evaluate a possible synergic effect with BZ. Using the combination of 1.25 µM TS and 1.0 µM BZ, a 46.66% presence of parasites was observed. By contrast, 79.14% of parasites were observed with TS alone at the same concentration. Similar results were observed with 2.5 µM TS and 1.0 µM BZ because 39.38% of parasites were found versus 58.57% with TS alone. There were no significant differences comparing 5 and 10 µM TS alone or with BZ (**Figure 6**).

## Discussion

There are no effective vaccines or drugs against T. cruzi, and the available drugs (NF and BZ) have low efficacy to treat the chronic phase of this disease and cause several side effects (Lascano et al., 2020). With the purpose to find new therapeutic targets, metabolic and defense pathways in *T. cruzi* have been studied in recent years. The detoxification of ROS is one of the main mechanisms implicated in the survival of the parasite, and for this reason, antioxidant enzymes have been considered as good therapeutic targets (González-Chávez et al., 2015).

The TcMPX have received particular attention due to their role as partner of several important proteins for cellular metabolism, as well as a virulence factor by protecting *T. cruzi* against NF effect, macrophage-derived peroxynitrite, and avoiding redox imbalance by ROS during mitochondrial energy generation (Piacenza et al., 2008; Piacenza et al., 2009; Peloso Ede et al., 2011; Piacenza et al., 2013; Peloso et al., 2016; Specker et al., 2022). In this study, the TcMPX from Querétaro and Ninoa Mexican *T. cruzi* strains were sequenced and analyzed *in silico*. The phylogenetic analysis showed that they are highly conserved proteins among *T. cruzi* strains, which suggests that variations in the sequence could affect their function and compromise the viability of *T. cruzi*. In addition, TcMPX is a single copy gene (Yeo et al., 2011). These reasons make it an attractive therapeutic target. However, to date, no inhibitor has been reported for this protein.

When comparing the sequence from QroMPX with other PRX previously characterized, we find that it shares 57% similarity with mitochondrial peroxidase (TXNII) reported in *T. cruzi* with which it interacts in oxidative stress conditions (Dias et al., 2018). Although both QroMPX and TXNII are mitochondrial, their similarity percentage is lower than that between QroMPX and human PRX2 and PRX3. This explains why homology modeling pointed to human PRX3 as temperate.

In addition, it was observed that the similarity is higher with MPX from other trypanosomatids (*L. brasiliensis* or *T. rangeli*) and human PRX3. This similarity of more than 60% in the sequence and highly similar spatial disposition of the QroMPX 3D model with PRX3 could suggest a similar function for both proteins, like an increase in its expression during heat stress events as has been shown for human PRX3 (Tchouagué et al., 2019). Although specific analysis showed that Qro MPX is unlikely to be a transmembrane protein, the presence of a sequence that has been proposed as a transmembranal domain in other PRXs opens the possibility that this protein may be secreted by *T. cruzi* or actively transported to the extracellular environment, probably to counteract ROS produced by the host, as proposed for other PRXs of trypanosomatids (Cuervo et al., 2009; Gadelha et al., 2013). It has been proposed that the PRX from *B. xylophylus* could use this motif to be transported across the intracellular membranes by ABC transporters (Li et al., 2011). This possibility cannot yet be ruled out for QroMPX and should be explored in the future. The possibility that this protein can be transported across the mitochondrial membrane or interact with it during cellular events has not been described and still needs to be explored as reported for other PRXs (Sasagawa et al., 2001; Choi et al., 2005). However, for the moment, we propose that it could be in soluble form as other mitochondrial and chloroplast PRXs (Flohé and Harris, 2007).

In the QroMPX sequence, the presence of GGLG and YF motifs was observed, which have been associated with the susceptibility of some PRXs to be hyper oxidated, leading to their inactivation (Cox et al., 2009). The QroMPX reported in this study is the first to be reported in *T. cruzi* with the presence of these structural motifs.

In addition, the molecular modeling showed that QroMPX monomers could interact by its β7-sheets, forming an interface type B to generate homodimers, as it was exemplified for other 2-cys PRXs (Poole and Nelson, 2016). In these structures, the C_P_ (C81) from a monomer could be associated with the C_R_ (C204) from the adjacent monomer. In this conformational structure, the arrangement of the GGLG and YF motifs and the catalytic triad (P74, T78, and R157) around the catalytic site are very similar to the human PRX3. This would facilitate the hyper oxidation of the molecule, but the presence and position of the amino acids N205 and P211 close to C_R_ (C204) indicate that QroMPX could be partially resistant to this phenomenon because the resistance to over-oxidation events in PRX3 is given by the presence of four amino acids (NTDP) in a similar position close to the C_R_ (Cox et al., 2009). Homology modeling also suggests that dimers can assemble into oligomers.

On other hand, several PRXs from protozoa have been proposed as therapeutic targets using molecules that inhibit it, which leads to a reduction in the resistance to oxidative stress (Haraldsen et al., 2009; König et al., 2011). Because of the similarity between QroMPX and PRX3, as well as the arrangement of the active site cysteines and the amino acids that surround them, it is very probable that QroMPX will be susceptible to the same inhibitors as PRX3. This feature can be used to reposition molecules that are currently therapeutic in humans. Several iron chelator molecules such as triapine, auranofin, and cisplatin reduce the presence of human PRX3 and have a pharmacological use in the treatment of some types of cancer (Myers, 2016). It would be interesting to test whether these molecules affect the *T. cruzi* proliferation or viability. Another molecule is TS, an excellent inhibitor of PRX3 that interacts with the active site cysteines, and this molecule also induces an oxidative stress condition in several types of cancer cells and is used as a treatment to reduce cell growth (Nelson et al., 2021). TS and some derivatives have shown good activity against protozoa as *Babesia* and *Plasmodium* sp. but have not been tested against other protozoa such as *T. cruzi* (Aminake et al., 2011; Aboulaila et al., 2012).

Docking analysis suggested that TS could bind to the resolutive-cysteine ​​in the catalytic site, but we do not rule out the possibility that both cysteines are involved because the functional unit of PRX is dimeric in a biological environment (Poole and Nelson, 2016). Likewise, because of the similarity found in the amino acid sequence (>55%) between QroMPX and TXNII, it is highly probable that TS also interacts with this protein, and the inhibition of both PRXs could be related to a synergistic effect.

When we incubated epimastigotes of *T. cruzi* for 48 h in the presence of TS, a reduction in the multiplication of the parasite in the culture was observed, without obvious morphological changes in the remaining parasites. In addition, cell-derived trypomastigotes and blood trypomastigotes (both infective) were susceptible to the trypanocidal effect of TS, but in a shorter time and with a lower drug concentration. At 24 h of incubation with different concentration of TS, no significant differences with respect to control groups were observed in intracellular amastigotes. Longer incubation time periods and combination with other drugs should be investigated in future works.

Previously, it has been shown that disrupting the electron transport chain in *T. cruzi* epimastigotes inhibits growth (Aldunate et al., 1986). The interaction between TS and QroMPX could interrupt the electron transport chain involved in the mitochondrial antioxidant network, leading to growth inhibition. On the other hand, it was very important to observe that *T. cruzi* trypomastigotes are more susceptible to the trypanocidal effect of TS because they are the ones that infect mammalian hosts, including humans. It has previously been shown that trypomastigotes from several strains of *T. cruzi* have two to six times more TcMPX than epimastigotes, so it has been proposed that it is one of the important enzymes for maintaining redox balance during energy generation in the infective stage (Piacenza et al., 2009; Gadelha et al., 2013). In addition, it has been reported that mitochondrial activity is greater in trypomastigotes than in epimastigotes, with the consequent production of H_2_O_2_ (Gonçalves et al., 2011). Because of this abundance of the protein and its biological role, it would be expected that when it is inhibited by TS, the effects would be faster and stronger on trypomastigotes than epimastrigotes, which is consistent with the IC_50_ differences observed.

TS IC_50_ values for cell-derived trypomastigotes (4.5 μM) and blood trypomastigotes (11 μM) are lower than those concentration used for healthy human cells. We observed that the IC_50_ of TS on Vero cells is greater than 40 μM, which is higher than the solubility limit of the compound. Similar results have been reported for human epidermal melanocytes at 24 h (Qiao et al., 2012).

TS can be used in combination with the classical treatment against Chagas disease. We compare the synergic effect of TS and BZ in trypomastigotes to reduce the concentration of both compounds, and we observed a significant reduction in the parasite number with the lowest TS-BZ combination used. Intriguingly, at high doses, we do not find synergic effect; further work should be done to address this phenomenon.

Therefore, the results obtained are promising, and they could be improved by making modifications to TS to make it more specific to PRX or by searching for similar molecules with higher selectivity.

Furthermore, the possibility of using TS in an animal model of infection with *T. cruzi*, as well as the mechanism of action, should be explored. In addition, the repositioning of this molecule, which is currently used in the human treatment of some oncological diseases, can be an alternative for a better Chagas disease treatment.

## Conclusions

The results reported in the present work suggest that TcMPX from *T. cruzi* has a high similarity with human PRX3, so this characteristic could be exploited to evaluate its susceptibility to inhibitors and their derivatives that are currently used for therapeutic purposes, such as TS that was found that reduced the growth of the parasite by a mechanism that could involve the change in the mitochondrial metabolism of *T. cruzi*.

## Data Availability Statement

The datasets presented in this study can be found in online repositories. The names of the repository/repositories and accession number(s) can be found in the article/**Supplementary Material**.

## Ethics Statement

The animal study was reviewed and approved by the Comite para el Cuidado y Uso de Animals de Laboratorio following the recommendation of the Ethical Code of the Instituto de Investigaciones Biomédicas, UNAM (https://www.biomedicas.unam.mx/wp-content/pdf/intranet/reglamentos/codigo-etico-iibo.pdf?x88126).

## Author Contributions

LR-S, IM, and BE designed the experiments. LR-S and RA-O sequenced the proteins. LR-S, PD-G, and IM performed homology modeling and bioinformatic analysis. LR-S and RC-H performed the molecular docking. LR-S and IM carried out the trypanocidal activity assays. LR-S, IM, and BE wrote the manuscript. All authors contributed to the article and approved the submitted version.

## Funding

BE acknowledges the financial support of DGAPA-PAPIIT, UNAM-IN206620, NUATEI-IIB 2019-2021, and CONACYT(CB) 160671.

## Conflict of Interest

The authors declare that the research was conducted in the absence of any commercial or financial relationships that could be construed as a potential conflict of interest.

## Publisher’s Note

All claims expressed in this article are solely those of the authors and do not necessarily represent those of their affiliated organizations, or those of the publisher, the editors and the reviewers. Any product that may be evaluated in this article, or claim that may be made by its manufacturer, is not guaranteed or endorsed by the publisher.
