# Supplementary figures and images for: Structural New Data for Mitochondrial Peroxiredoxin From Trypanosoma cruzi Show High Similarity With Human Peroxiredoxin 3: Repositioning Thiostrepton as Antichagasic Drug
Source: Front Cell Infect Microbiol. 2022 Jul 6;12:907043. doi: 10.3389/fcimb.2022.907043 (PMC9301493; doi:10.3389/fcimb.2022.907043)

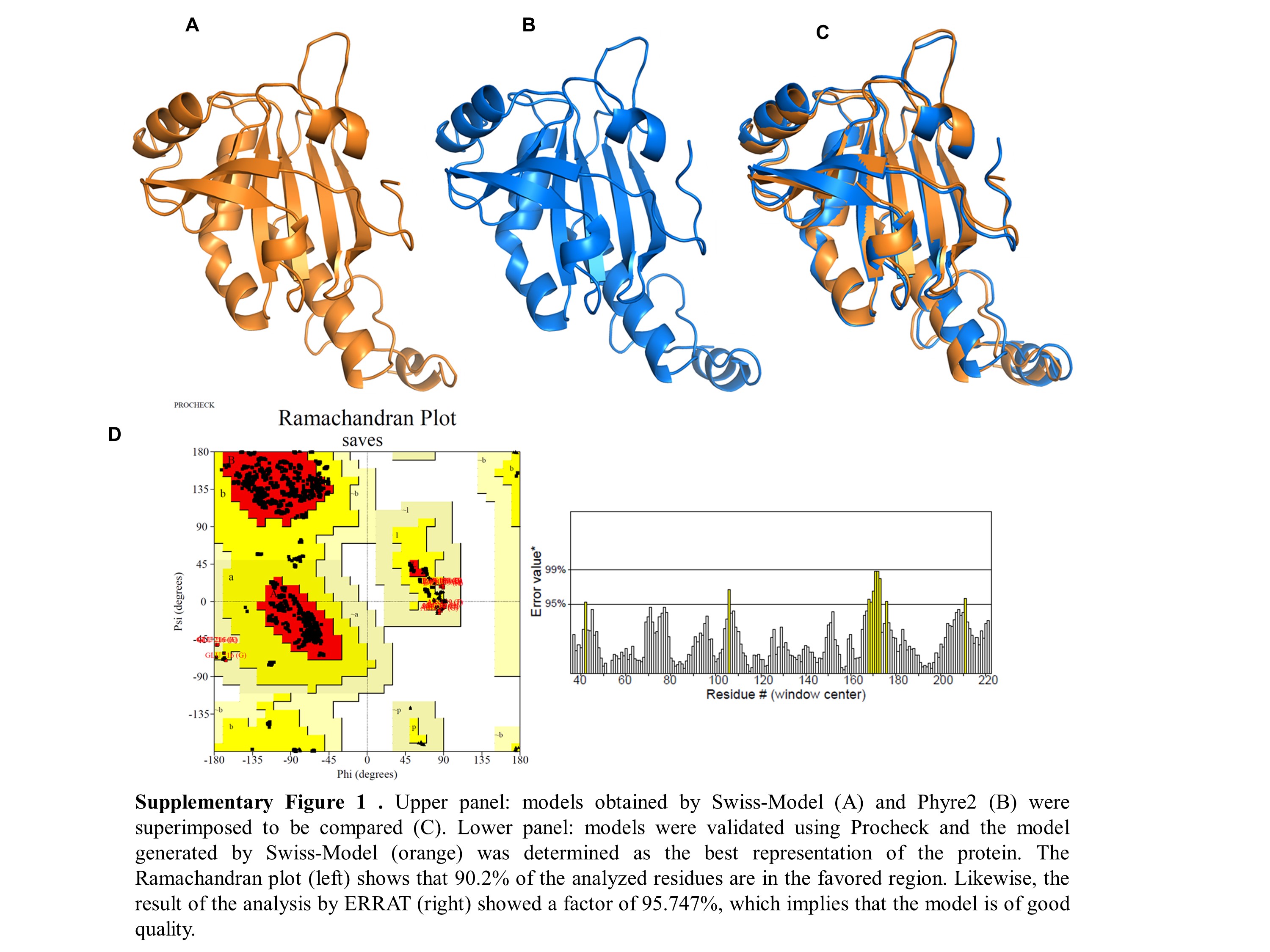

Supplement: Supplementary file 1 [file Image_1.jpeg]

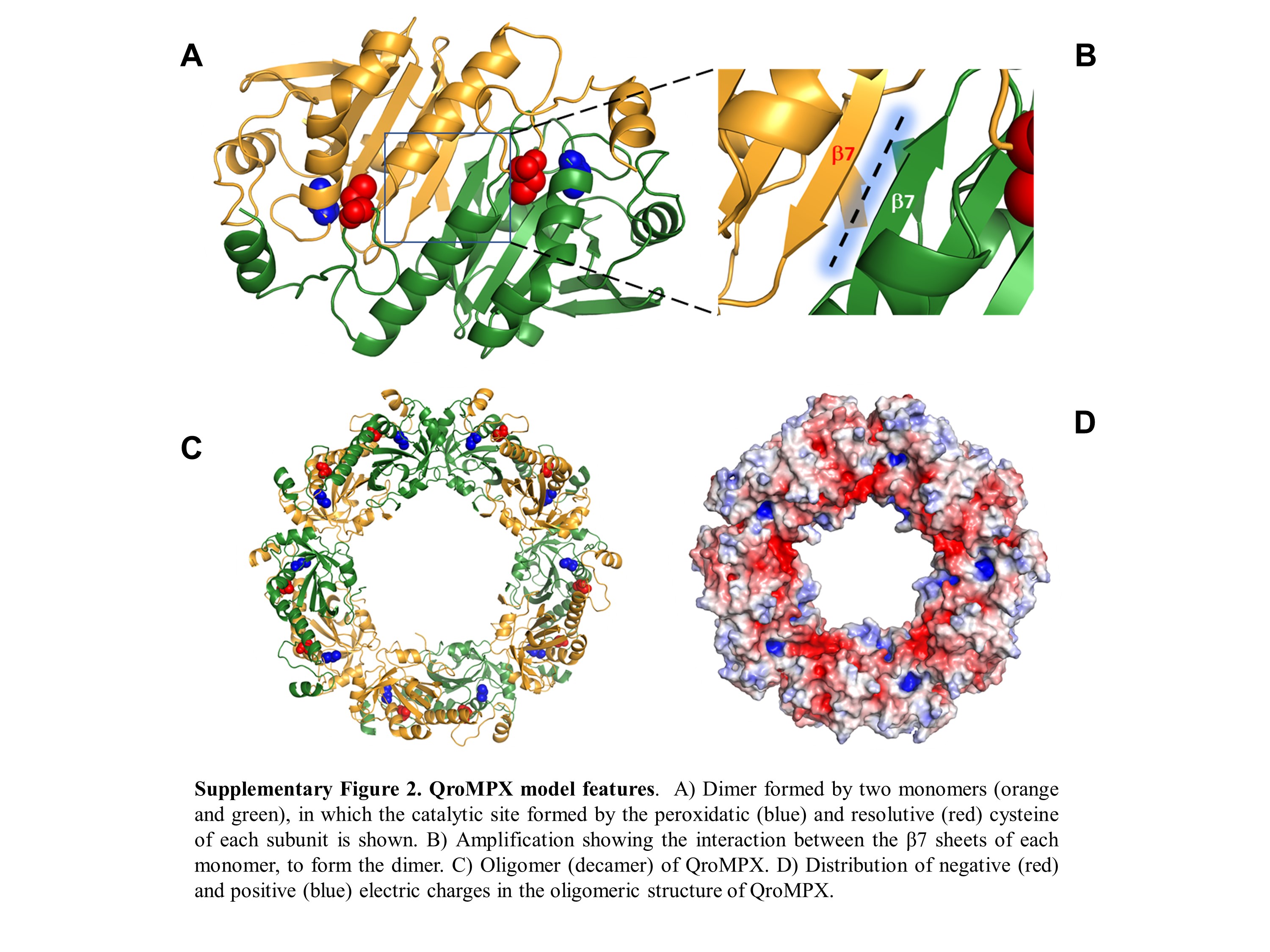

Supplement: Supplementary file 2 [file Image_2.jpeg]

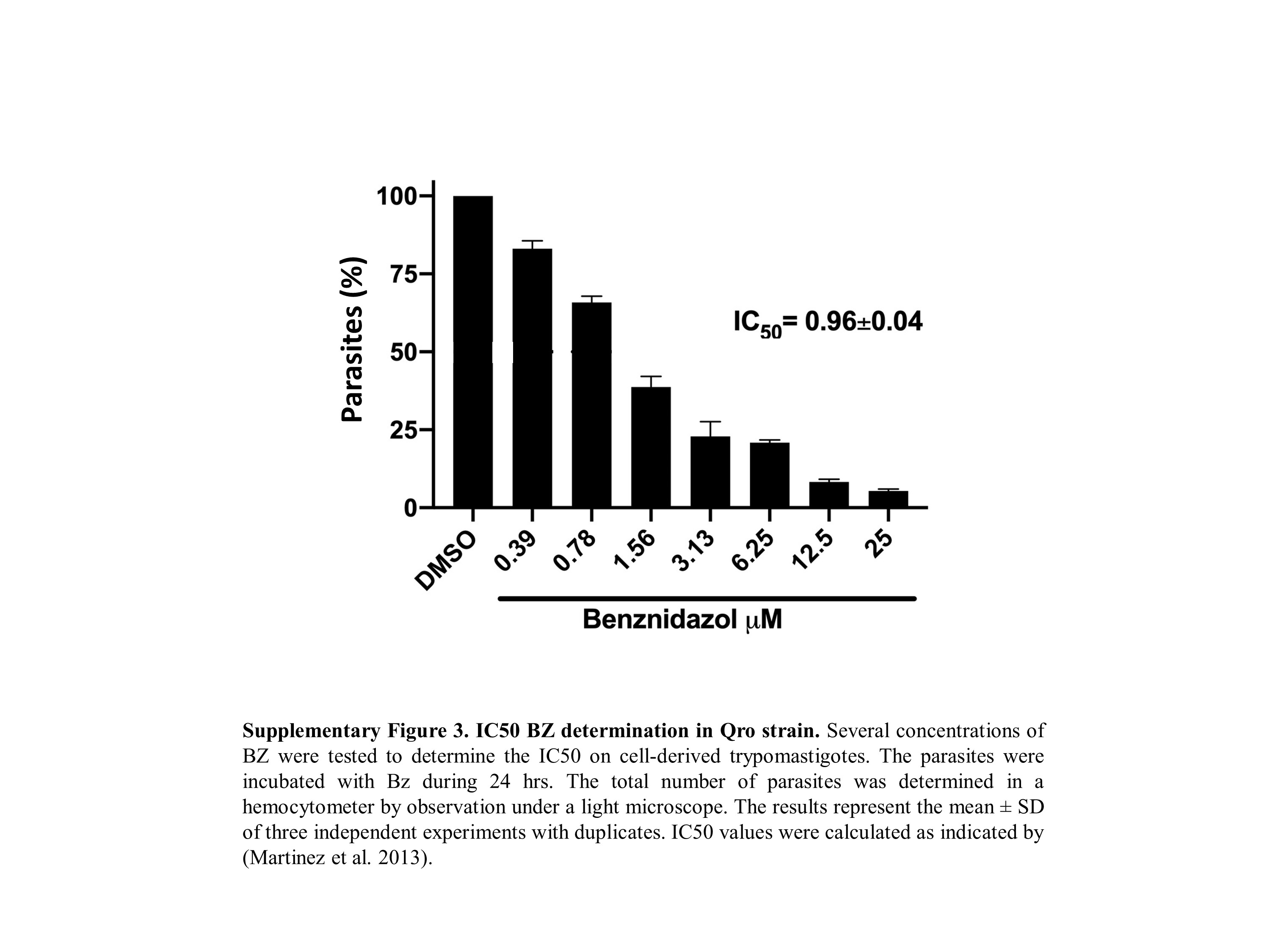

Supplement: Supplementary file 3 [file Image_3.jpeg]
