# Supplementary material for: Structural New Data for Mitochondrial Peroxiredoxin From Trypanosoma cruzi Show High Similarity With Human Peroxiredoxin 3: Repositioning Thiostrepton as Antichagasic Drug
Source: Front Cell Infect Microbiol. 2022 Jul 6;12:907043. doi: 10.3389/fcimb.2022.907043 (PMC9301493; doi:10.3389/fcimb.2022.907043)
Supplement: Supplementary file 4 [file Table_1.docx]

**Supplementary Table 1.** Mitochondrial peroxiredoxins sequences used in this work

| **Species** | **DTU*** | **Name** | **Accession number **** |
| --- | --- | --- | --- |
| *Trypanosoma cruzi* | I | Silvio X10cl1 | AIB07568.1 |
|  |  | Qro | QKE53459.1 |
|  |  | Ninoa | QKE53460.1 |
|  | II | Esmcl3 | AIB07581.1 |
|  | III | X109/2 | AIB07585.1 |
|  | IV | 92122102R | AIB07575.1 |
|  | V | MNcl2 | AIB07586.1 |
|  |  | Sc43cl1 | AIB07589.1 |
|  | VI | CL Brener | AIB07590.1 |
|  |  | Tulahuencl2 | AIB07592.1 |
| *Trypanosoma rangeli* |  | SC58 Cont6481 | ESL05855.1 |
| *Trypanosoma conorhini* |  | Mitochondrial PRX | RNF02014.1 |
| *Angonomas deanei* |  | Mitochondrial PRX | EPY42814.1 |
| *Strigomonas culicis* |  | Mitochondrial PRX | EPY22216.1 |
| *Leishmania amazonensis* |  | Mitochondrial PRX | AAX47429.1 |
| *Leishmania tropica* |  | Mitochondrial PRX | AYV64378.1. |
| *Leishmania donovani* |  | Mitochondrial PRX | AAX73294.1 |
| *Leishmania brasiliensis* |  | Mitochondrial PRX | XP_001562236.1 |
| *Plasmodium falciparum* |  | Mitochondrial PRX | PDB-2CODA |
| *Plasmodium falciparum* |  | Mitochondrial PRX | PDB-2CODB |
| *Candida albicans* |  | Mitochondrial PRX | EEQ47073.1 |
| *Bursaphelenchus xylophilus* |  | 2-cysteine peroxiredoxin | ABW81468.1 |
| *Toxoplasma gondii* |  | Peroxiredoxin | AAG25678.2 |
| *Homo sapiens* |  | Peroxiredoxin 3 (PRDX3) | ABB84468.1 |
|  |  | Peroxiredoxin 3 (PRDX3) | PDB-5JCG |
|  |  |  |  |

*Only for *T. cruzi*

**From GenBank, except PDB: Protein Data Bank.
